# Prevalence of Metabolic Syndrome and Its Association With Clinical Correlates and Caregiver Burden in Patients With Bipolar Disorder at a Tertiary Hospital

**DOI:** 10.7759/cureus.72211

**Published:** 2024-10-23

**Authors:** Prince Antony, Neethu S, Kathleen A Mathew, Bindu Menon

**Affiliations:** 1 Psychiatry, Amrita Institute of Medical Sciences and Hospital, Kochi, IND

**Keywords:** bipolar disorder, caregiver burden, metabolic syndrome, suicidality

## Abstract

Introduction: Metabolic syndrome is a cluster of metabolic abnormalities, including obesity, insulin resistance, dyslipidemia, and hypertension. The prevalence of metabolic syndrome is recognized to be high among patients with bipolar disorder. This study is relevant given the limited number of Indian studies exploring the prevalence of metabolic syndrome and its association with clinical correlates in bipolar patients. This research aims to inform clinicians about the need for timely interventions in diagnosing and managing metabolic syndrome in this population, which may contribute to significant morbidity and poor clinical outcomes.

Methods: A cross-sectional study was conducted in the inpatient and outpatient units of the psychiatry department involving 83 subjects diagnosed with bipolar disorder. Informed consent was obtained, and sociodemographic and clinical data were collected. The National Cholesterol Education Program (NCEP)-Adult Treatment Panel III (ATP III) criteria were used to diagnose metabolic syndrome. The Young Mania Rating Scale (YMRS) and Hamilton Depression Rating Scale (HAM-D) were applied to assess the severity of manic and depressive episodes, respectively. The Suicide Behaviors Questionnaire-Revised (SBQ-R) was used to assess suicidal risk, and the Burden Assessment Schedule (BAS) was utilized to evaluate caregiver burden. Statistical significance was tested using the independent-sample t-test and Mann-Whitney U test for continuous variables, along with the chi-square test for categorical variables. A p-value of <0.05 was considered significant.

Results: Metabolic syndrome was noted in 49 (59%) patients with bipolar disorder. Subjects with metabolic syndrome exhibited more severe manic episodes, had a higher number of lifetime episodes, were more likely to have a history of suicide attempts, and showed increased suicidal risk. Patients on polypharmacy were at higher risk of developing metabolic syndrome. Caregivers of patients with metabolic syndrome reported greater caregiver burden compared to those without the condition.

Conclusion: There is a high prevalence of metabolic syndrome in patients with bipolar disorder. Metabolic syndrome is associated with adverse prognostic factors, including a higher number of lifetime episodes, greater severity of manic episodes, and increased suicidality. This underscores the necessity for routine monitoring of blood and anthropometric parameters.

## Introduction

Bipolar disorder is a chronic mental illness characterized by an episodic pattern and inter-episodic recovery. Evidence indicates that individuals with bipolar disorder have a significantly higher prevalence of cardiovascular risk factors, including obesity, insulin resistance, hypertension, and dyslipidemia, which together constitute metabolic syndrome [1]. Grover et al. [1] conducted a systematic review of 34 studies from various nations and ethnic backgrounds, reporting that metabolic syndrome prevalence rates in bipolar disorder range from 16.7% to 67%, significantly exceeding those in the general population. Most available data originate from Western studies, with limited research conducted within the Indian population.

Factors contributing to the increased risk of metabolic syndrome in bipolar patients include genetics, abnormal hypothalamic-pituitary-adrenal (HPA) axis activity, chronic low-grade inflammation, lifestyle factors such as sedentary behavior and unhealthy diets, substance use, and certain psychotropic medications [2-6]. Existing data suggest that the risk of metabolic syndrome increases with age, longer duration of illness and treatment, a greater number of lifetime episodes, the index episode being depression, more severe depressive episodes, higher age at the onset of illness, and the use of certain medications, particularly atypical antipsychotics and mood stabilizers like lithium, valproate, and carbamazepine [1,7].

Only a limited number of studies have systematically evaluated the association between clinical correlates of bipolar disorder and metabolic syndrome in the Indian context [7,8]. Data regarding the association between metabolic syndrome and suicidality are conflicting according to Western studies, and no Indian studies have explored this area [9]. Some evidence suggests that metabolic syndrome worsens clinical outcomes in bipolar patients [1]; therefore, it is important to assess whether comorbid metabolic syndrome poses a greater burden on caregivers. However, this area has not been adequately addressed in the literature. Our study seeks to address these gaps in the existing literature.

## Materials and methods

This was a cross-sectional observational study conducted in the Psychiatry department of a tertiary hospital. The study was conducted over a one-year period, from 2020 to 2021, on patients diagnosed with bipolar disorder. Patients admitted to the psychiatry ward during an episode of either mania or depression, as well as patients in remission receiving treatment in the outpatient unit of the Psychiatry department, who met the inclusion and exclusion criteria and provided informed consent, were included in the study. The sample was collected using a purposive sampling method. The inclusion criteria were patients aged 18 years or older who fulfilled the diagnostic criteria for bipolar disorder based on the ICD-10 (International Classification of Diseases-10). The exclusion criteria consisted of patients with significant cognitive impairment.

Based on the proportion of metabolic syndrome observed in an earlier publication [3], with 20% relative precision and 95% confidence limits, the minimum sample size was determined to be 83. Upon obtaining approval from the Institutional Review Board, the study was commenced. A total of 83 patients diagnosed with bipolar disorder, meeting the inclusion and exclusion criteria and providing written informed consent, were included in the study.

Assessments

A semi-structured data collection form was used to record participant and caregiver data. The Modified National Cholesterol Education Program Adult Treatment Panel III (NCEP ATP III) guidelines [10] were adopted for diagnosing metabolic syndrome. According to the NCEP ATP III criteria, metabolic syndrome is present when three or more of the following five criteria are met: increased waist circumference (greater than 102 cm for men and greater than 88 cm for women), elevated fasting triglycerides (≥150 mg/dL or on treatment), low high-density lipoprotein (HDL) levels (less than 40 mg/dL in men and less than 50 mg/dL in women or on treatment), high blood pressure (≥130/85 mm Hg or on treatment), and elevated fasting blood glucose (greater than 100 mg/dL or on treatment).

The Young Mania Rating Scale (YMRS) was used to assess the severity of manic episodes. It consists of 11 items based on the core symptoms of mania. Four items are graded on a 0-8 scale, and the remaining seven items are graded on a 0-4 scale [11]. The Hamilton Depression Rating Scale (HDRS) was used to assess the severity of depressive episodes. This scale contains 17 items that assess depressive symptoms experienced by the patient over the past week. A score of 0-7 indicates clinical remission, while a score of 20 or higher suggests a moderate to severe depressive episode [12].

The Suicidal Behaviors Questionnaire-Revised (SBQ-R), developed by Osman et al., was used to assess suicidality [13]. This self-reported, four-item questionnaire covers four constructs within the suicidal behavior domain: lifetime suicidal ideation and attempts, frequency of suicidal ideation over the past year, threats of suicide, and self-assessed probability of future suicidal behavior. These items are assessed on a Likert scale, resulting in a total score ranging from 3 to 18. A score of 8 or higher indicates a significant risk of suicidal behavior.

The Burden Assessment Schedule (BAS) was used to assess caregiver burden. This 20-item questionnaire, published by the WHO in 1998, evaluates five main factors: impact on well-being, quality of marital relationships, appreciation for caregiving, impact on relationships with others, and perceived severity of the disease [14].

Statistical analysis

IBM SPSS Statistics for Windows, Version 20 (Released 2011; IBM Corp., Armonk, New York) was used for statistical analysis. Frequencies and percentages were calculated to express categorical variables, while the mean and standard deviation were used to describe continuous variables. An independent-sample t-test was used to determine the statistical significance of differences in continuous variables for normally distributed data, and the Mann-Whitney U test was applied for non-normally distributed data. The chi-square test was used to assess the statistical significance of the association between categorical variables and metabolic syndrome. A p-value of less than 0.05 was considered indicative of a statistically significant association.

## Results

In our study, a total of 83 patients with bipolar disorder were included, of which 65 (78.3%) were in a manic episode, 11 (13.3%) were in a depressive episode, and 7 (8.4%) were in remission. The mean age of the study population was 41.55 ± 16.453 years. Among the participants, 38 (45.8%) were in the age group of 20-35 years, 17 (20.5%) were in the age group of 36-50 years, and 28 (33.7%) were in the age group over 50 years. Of the study population, 37 (44.6%) were male, while 46 (55.4%) were female. Additionally, 20 (24.1%) had the highest educational qualification of less than 12th grade, 24 (28.9%) had completed 12th grade, and 39 (47%) were graduates, diploma holders, or postgraduates. Furthermore, 44 (53%) were unemployed, 13 (15.7%) were professionals, and 26 (31.3%) were employed in manual labor.

**Figure 1:**
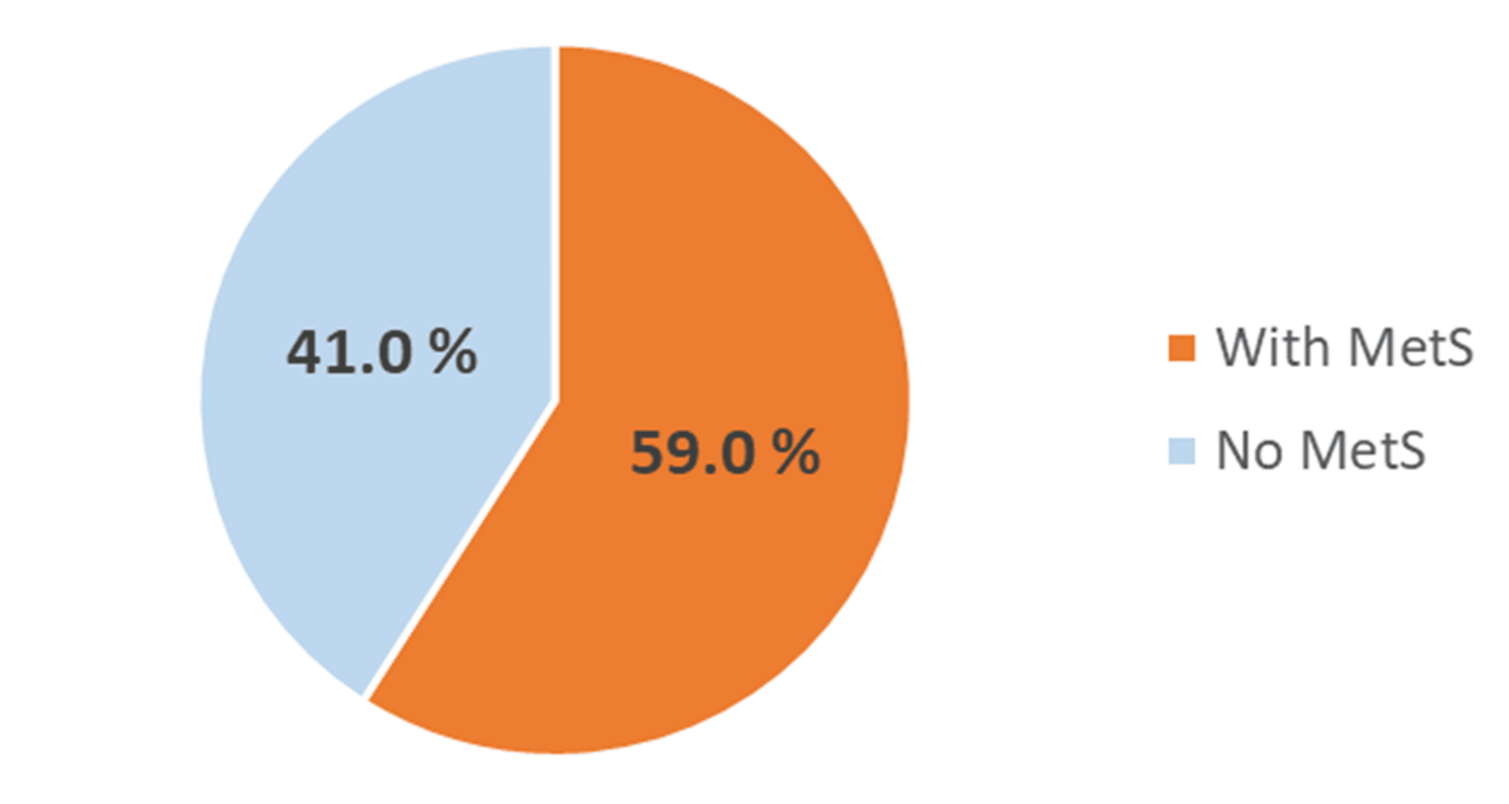
Prevalence of metabolic syndrome MetS: metabolic syndrome.

The prevalence of metabolic syndrome among bipolar affective patients in this study was found to be 59% (n = 49, 95% confidence interval (CI): 48.4% to 69.6%) (Figure 1).

**Figure 2:**
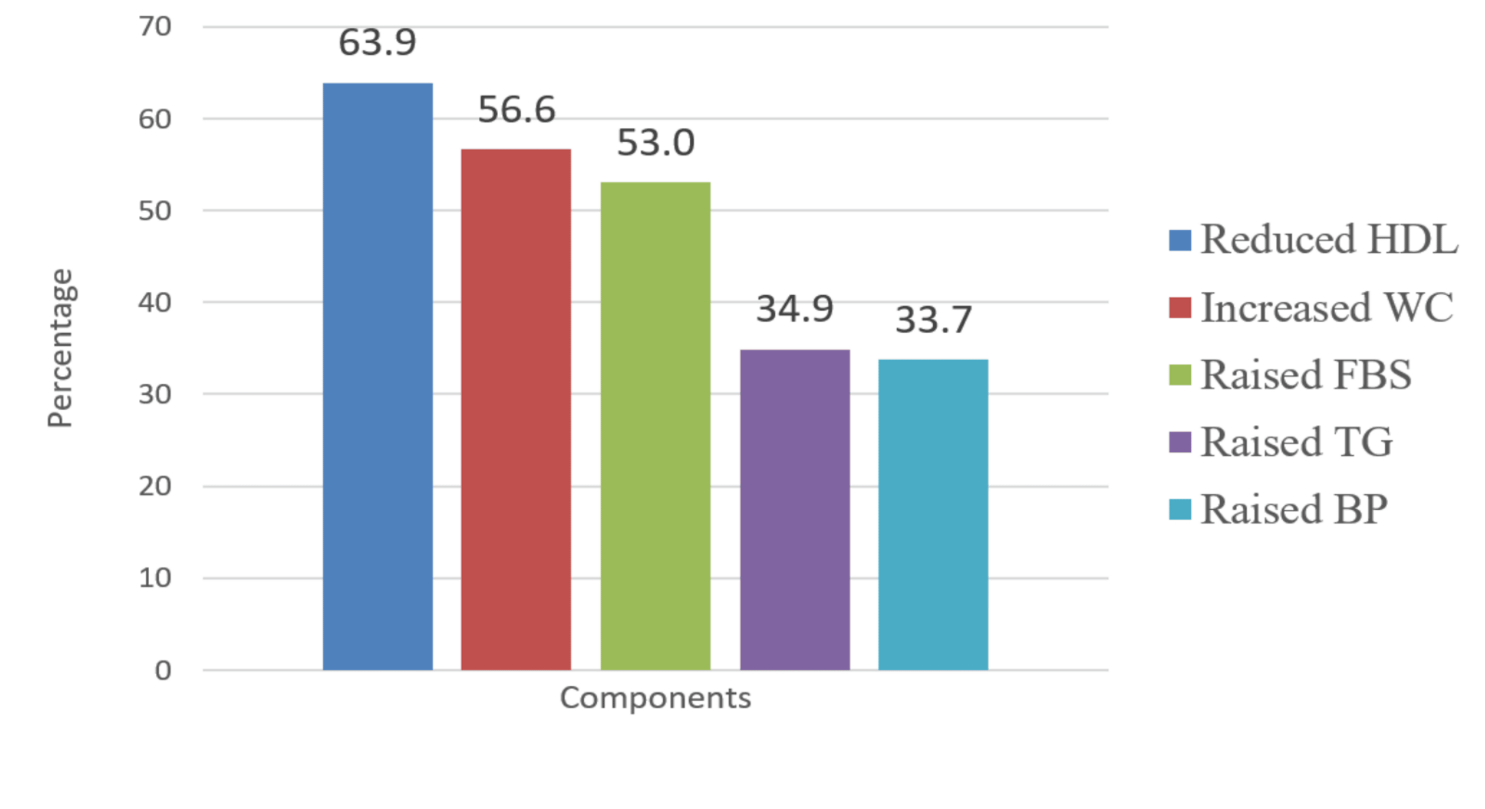
Distribution of components of metabolic syndrome HDL: high-density lipoprotein, WC: waist circumference, FBS: fasting blood sugar, TG: triglycerides.

Reduced HDL was the most commonly deranged metabolic parameter, noted in 53 (63.9%) patients, followed by increased waist circumference, observed in 47 (56.6%) patients. The least commonly noted abnormality was elevated blood pressure, recorded in only 28 (33.7%) patients (Figure 2).

The mean age of bipolar patients with metabolic syndrome was found to be significantly higher compared to those without metabolic syndrome (p < 0.001). No statistically significant association was found between other sociodemographic variables and metabolic syndrome.

An increased prevalence of metabolic syndrome was noted in patients with a higher age at the onset of illness (p = 0.003), longer duration of illness (p < 0.001), longer duration of treatment (p < 0.001), a greater number of total lifetime episodes (p = 0.001), and a greater number of manic episodes (p = 0.007); all these associations were statistically significant. No statistically significant association was observed between the index episode and metabolic syndrome.

Caregiver burden was assessed using the Burden Assessment Schedule (BAS). A statistically significant association was found between caregiver burden and metabolic syndrome, with caregivers of patients with metabolic syndrome having a higher mean BAS score compared to those without metabolic syndrome (p < 0.001) (Table 1).

**Table 1:** Association of age and continuous clinical variables with metabolic syndrome *p < 0.05 is significant. MetS: metabolic syndrome, BAS: Burden Assessment Schedule.

Variable	With MetS (Mean ± SD)	Without MetS (Mean ± SD)	U	p
Age	48.84 ±15.222	31.06 ± 11.972	270.00	<0.001*
Age at onset of illness	30.49 ± 11.721	24.32 ± 8.892	516.5	0.003*
Duration of illness	18.22 ±12.135	6.76 ± 5.433	324	<0.001*
Total episodes	6.9 ± 5.229	3.59 ± 1.925	470.5	0.001*
Number of manic episodes	4.98 ± 4.474	2.47 ±2.048	546.5	0.007*
Number of depressive episodes	1.80 ± 3.279	1.00 ±1.015	804.5	0.781
Duration of treatment	12.37± 8.715	3.76 ± 3.718	301.5	<0.001*
Mean BAS score	34.57 ±10.169	23.82 ± 6.389	349	<0.001*

**Figure 3:**
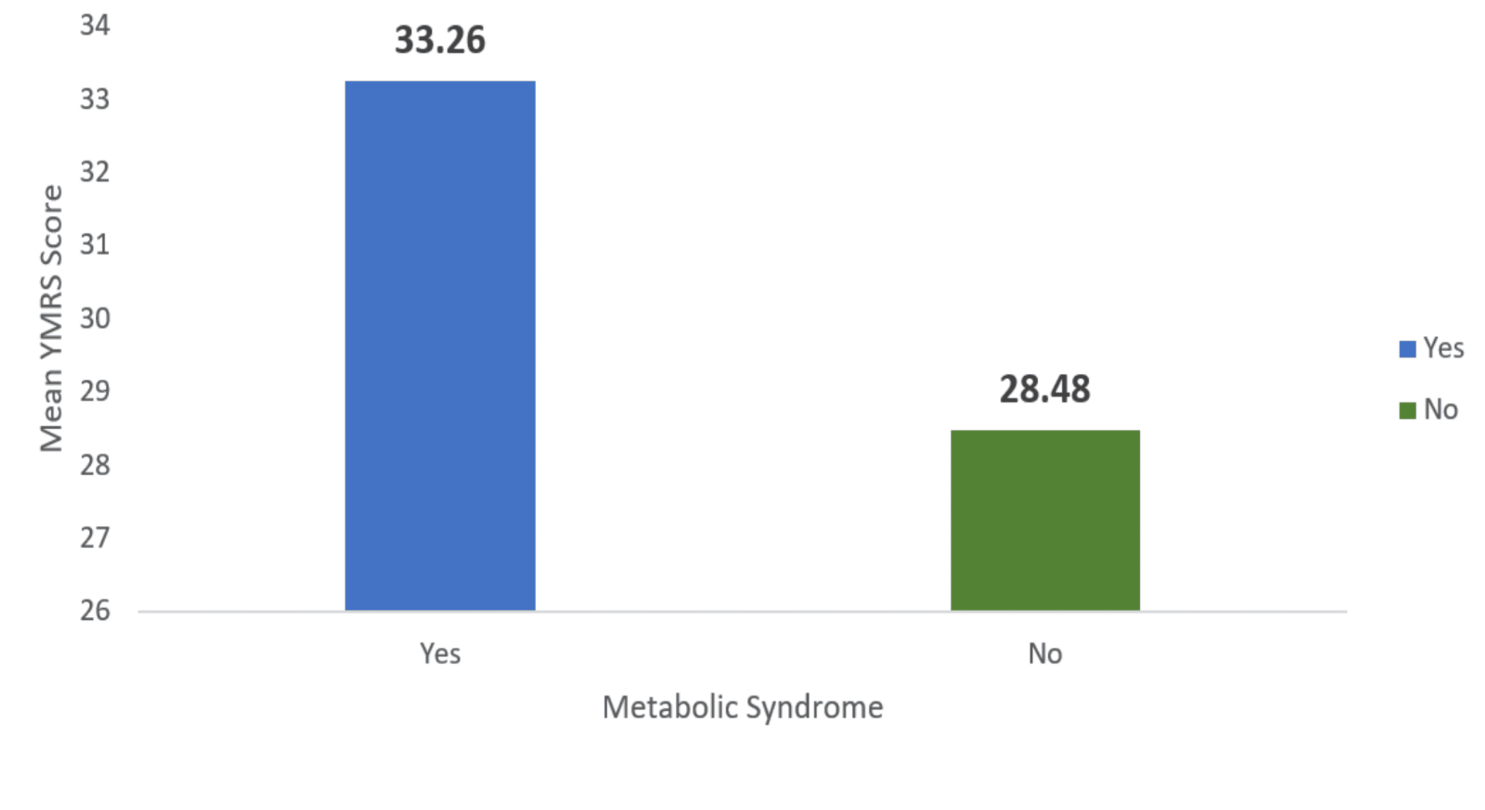
Comparison of severity of manic episode in patients with and without metabolic syndrome YMRS: Young Mania Rating Scale.

Those with metabolic syndrome had a higher mean YMRS score (33.26 ± 9.717) compared to those without metabolic syndrome (28.48 ± 9.019) (p = 0.048) (Figure 3). No statistically significant association was found between metabolic syndrome and the severity of depressive episodes.

Patients with a higher suicidal risk, as indicated by an SBQ-R score ≥ 8, had a higher prevalence of metabolic syndrome compared to patients with an SBQ-R score < 8 (p = 0.002). Additionally, patients with a history of suicide attempts had a higher prevalence of metabolic syndrome compared to those without such a history (p = 0.026).

Medications found to have a statistically significant association with the development of metabolic syndrome included lithium, sodium valproate, and risperidone. An increased prevalence of metabolic syndrome was noted in patients on two mood stabilizers (p = 0.001), one mood stabilizer plus an antipsychotic (p < 0.001), and those on more than two medications (p < 0.001), compared to those who were not. All these associations were statistically significant (Table 2).

**Table 2:** Association of categorical clinical variables with metabolic syndrome *p < 0.05 is significant. MetS: metabolic syndrome, MS: mood stabilizer, AP: antipsychotic.

Variable		With MetS n (%)	Without MetS n (%)	Chi-square	p
Index episode	Mania	31 (66)	16 (34)	2.146	0.143
	Depression	18 (50)	18 (50)		
H/o suicidal attempts	Present	12 (85.7)	2 (14.3)	4.956	0.026*
	Absent	37 (53.6)	32 (46.4)		
Suicidal risk	SBQ-R<8	32 (50)	32 (50)	9.44	0.002*
	SBQ-R≥8	17 (89.5)	2 (10.5)		
Lithium	Yes	27 (79.4)	7 (20.6)	9.887	0.002*
	No	22 (42.9)	27 (55.1)		
Sodium valproate	Yes	28 (73.7)	10 (26.3)	6.219	0.013*
	No	21 (46.7)	24 (53.3)		
Clozapine	Yes	4 (100)	0 (0)	1.408	0.235
	No	45 (57)	34 (43)		
Olanzapine	Yes	14 (70)	6 (30)	1.31	0.252
	No	35 (55.6)	28 (44.4)		
Risperidone	Yes	15 (78.9)	4 (21.1)	4.04	0.044*
	No	34 (53.1)	30 (46.9)		
At least 1 MS + AP	Yes	40 (80)	10 (20)	22.85	<0.001*
	No	9 (27.3)	24 (72.7)		
At least 2 MS	Yes	22 (84.6)	4 (15.4)	10.243	0.001*
	No	27 (47.4)	30 (52.6)		
More than 2 medications	Yes	33 (84.6)	6 (15.4)	19.904	<0.001*
	No	16 (36.4)	28 (63.6)		

## Discussion

This study was conducted with limited background evidence indicating that the proportion of patients with metabolic syndrome in bipolar disorder is high, based on studies conducted in Western countries [15]. Despite increasing rates of early-onset diabetes mellitus and hypertension in India, few studies have assessed the association between bipolar disorder and metabolic syndrome in the Indian context. After obtaining informed consent, we selected 83 patients diagnosed with bipolar affective disorder according to ICD-10 criteria, using purposive sampling. These patients sought treatment in the Psychiatry department of a tertiary care center in Kerala. Of the total study subjects, 65 (78.3%) were in a manic episode, 11 (13.3%) were in a depressive episode, and 7 (8.4%) were in remission. The mean age of the study subjects was 41.55 ± 16.453 years.

In our study, the prevalence of metabolic syndrome was as high as 59%, which falls within the range reported in a review by Grover et al. [1]. In this review of 34 studies, the prevalence of metabolic syndrome ranged from 16.7% to 67% across different countries. The sample sizes of these studies varied from 15 to 822 participants. This wide range of prevalence could be attributed to differences in the criteria used to diagnose metabolic syndrome, variations in sample sizes, cultural differences in dietary patterns, and variations in ethnicity. In a study conducted in North India [8], which included 200 patients, the prevalence was reported as 41% using the modified NCEP-ATP III criteria and 40% when assessed with the International Diabetes Federation criteria. In another study conducted in southern India, which included 67 subjects, more than half of the study population had metabolic syndrome, with a prevalence of 53.7%.

In our study, the prevalence of metabolic syndrome was higher compared to the aforementioned Indian studies. This could be because the study by Kumar et al. [7] excluded individuals with a history of diabetes, hypertension, and ischemic heart disease prior to the bipolar diagnosis and the initiation of medications. In our study, this subset of individuals was not excluded. Additionally, differences in eating habits and physical activity levels could be attributed to geographic and cultural variations.

In our study, reduced HDL was reported as the most common abnormal component, noted in 53 (63.9%) patients, followed by increased waist circumference in 47 (56.6%) subjects. In contrast, raised blood pressure was the least reported abnormality, noted in 28 (33.7%) subjects. In a review by Grover et al. [1], increased waist circumference and elevated blood pressure were identified as the most common abnormalities in 7 of 19 studies. Elevated fasting glucose was the least commonly reported abnormality, while lipid abnormalities had intermediate prevalence in the review. All components of metabolic syndrome in our study fell within the reported range, except for elevated fasting blood sugar (FBS), which was higher than that noted in Grover’s review. This discrepancy could be attributed to geographical variations in metabolic abnormalities. Reduced HDL was the most prevalent abnormality in our sample, consistent with the findings of Sicras et al. [16].

Patients with metabolic syndrome had a higher mean age compared to those without it, and this association was statistically significant (p = 0.001). This result was consistent with the findings of Kumar et al. [7]. These findings suggest that clinicians should exercise caution when prescribing psychotropic medications to older individuals and closely monitor metabolic parameters. The link between gender and metabolic syndrome was not significant in our study, which aligns with earlier studies [16-18] that found no association. Metabolic syndrome was significantly associated with a longer duration of illness (p < 0.001). Subjects with metabolic syndrome had a longer mean duration of illness compared to those without it, consistent with previous studies conducted in India and other countries by Chang et al. [19], Taylor et al. [20], and Salvi et al. [21].

Total lifetime episodes were found to have a significant association with metabolic syndrome (p = 0.001) in our study. Patients with metabolic syndrome had a greater number of lifetime episodes compared to those without it. This result is consistent with the Indian study by Kumar et al. [7] and the study by Fagiolini et al. [22]. This association could be attributed to the likelihood that as the number of episodes increases, there is greater immune and endocrine dysregulation, along with an increase in the dose of psychotropics during the acute phase for rapid symptom control, all of which can elevate the risk of metabolic syndrome. Additionally, the total number of lifetime manic episodes was significantly higher in patients with metabolic syndrome (p = 0.007). Unlike the findings of Kumar et al. [7], our study did not reveal any significant association between the number of lifetime depressive episodes and metabolic syndrome. This may be due to the lower mean total number of depressive episodes in our study, with only 1.47 ± 2.62 episodes.

Subjects with metabolic syndrome had a later age at the onset of illness compared to those without it (p = 0.003). These results align with a study conducted in Southern China by Guan et al. [23]. It was observed that patients with metabolic syndrome experienced more severe manic episodes, as indicated by a higher mean YMRS score compared to those without it, and this association was significant (p = 0.048). However, this finding contrasts with a South Indian study, which reported that patients with metabolic syndrome exhibited less severe manic symptoms. This discrepancy may be attributed to the possibility that patients with more severe manic episodes in our study were treated with a greater number of psychotropics, contributing to the metabolic burden. The severity of depressive episodes did not show any association with metabolic syndrome, consistent with the results of Kumar et al. [7].

In our study, 34 (41%) subjects were on lithium, and 38 (45.8%) were on sodium valproate as mood stabilizers. Additionally, 20 (24.1%) were on olanzapine, 19 (22.9%) on risperidone, and only 4 (4.8%) were on clozapine. Although it is not possible to establish a cause-effect relationship between metabolic syndrome and psychotropic medications, our study suggests that the use of certain medications, such as sodium valproate, lithium, risperidone, and polypharmacy during the maintenance phase, poses an additional risk of metabolic syndrome among the study subjects. The percentage of patients on lithium who had metabolic syndrome (27, 79%) was almost the same as those on sodium valproate (28, 73.7%). In our study, the use of antipsychotics like clozapine and olanzapine did not reach statistical significance, possibly due to the small sample size and the limited number of patients receiving these medications. Only four subjects were on clozapine, and all of them had metabolic syndrome. A statistically significant association was found between metabolic syndrome and the use of a greater number of medications, specifically the use of at least two mood stabilizers and at least one antipsychotic plus a mood stabilizer, consistent with the findings of Garcia-Portilla et al. [18] and Chang et al. [19].

There is very limited data on the association between metabolic syndrome and suicidality, and the results are conflicting. In our study, the association of both suicide attempts and suicidal risk with metabolic syndrome was statistically significant. Patients with a history of suicide attempts and high suicidal risk had a higher prevalence of metabolic syndrome. A similar association was noted by Fagiolini et al. [22], who reported that patients with metabolic syndrome were more likely to have a lifetime history of at least one suicide attempt compared to those without metabolic syndrome (53% vs. 36%), with a statistically significant association. This relationship may be explained by biological mechanisms, such as the effects of inflammation, oxidative stress, and endocrine dysregulation, which are heightened in the presence of metabolic syndrome and may contribute to the increased risk of suicidality.

However, conflicting evidence exists. Stenzel et al. [9] found no significant association between metabolic syndrome and suicidality in bipolar patients. This discrepancy highlights the need for further research to clarify the relationship between these factors and to understand the reasons for these differences across studies.

Caregiver burden is a significant clinical concern that is often not routinely addressed in busy clinical settings. Considering the evidence that bipolar patients with metabolic syndrome can experience adverse clinical outcomes, we also examined whether comorbid metabolic syndrome poses a greater burden on caregivers.

Our study indicated that caregivers of patients with metabolic syndrome experienced a higher caregiver burden, as evidenced by a higher mean BAS score compared to caregivers of patients without metabolic syndrome. The association between caregiver burden and metabolic syndrome was highly significant (p < 0.001). The high caregiver burden may be explained by various clinical factors related to the patient, such as a longer duration of illness, a higher number of lifetime suicide attempts, increased suicidal risk, and a greater number of medications, which require caregivers to manage more side effects and monitor medication adherence more frequently.

The strengths of our study include the assessment of the association between clinical variables, particularly suicidality and episode severity, which have not received adequate attention in previous studies. Our study is the first to assess whether metabolic syndrome confers additional caregiver burden among family members of these patients.

Limitations of our study include its cross-sectional design, which precludes establishing a cause-effect relationship, as well as the small sample size and the possibility of recall bias concerning treatment details and past episodes. The skewed distribution of the sample, with a predominance of manic states, may limit the generalizability of our findings, particularly regarding the relationship between the number and severity of depressive episodes and metabolic syndrome.

## Conclusions

Metabolic syndrome is highly prevalent among individuals with bipolar disorder and is associated with clinical variables such as prolonged illness duration, a higher number of lifetime mood episodes, a later age of illness onset, and polypharmacy. Patients with metabolic syndrome often experience more severe manic episodes and an increased risk of suicidality, suggesting that these metabolic comorbidities contribute to the overall clinical complexity of the disorder. Additionally, caregivers of bipolar patients with metabolic syndrome tend to experience a greater burden, likely due to the chronicity and severity of the illness in this population. Our findings underscore the need for early detection and targeted interventions for metabolic risk factors, as these measures may significantly influence the clinical trajectory of bipolar disorder by mitigating illness severity and improving clinical outcomes.
